# Mechanical Properties of Polyvinyl Alcohol Fiber-Reinforced Cementitious Composites after High-Temperature Exposure

**DOI:** 10.3390/gels8100662

**Published:** 2022-10-17

**Authors:** Peng Zhang, Peishuo Zhang, Jingjiang Wu, Yong Zhang, Jinjun Guo

**Affiliations:** 1 Yellow River Laboratory, Zhengzhou University, Zhengzhou 450001, China; 2 School of Water Conservancy Engineering, Zhengzhou University, Zhengzhou 450001, China; 3 Communications Construction Company of CSCEC 7th Division Co., Ltd., Zhengzhou 450004, China

**Keywords:** cementitious composites, polyvinyl alcohol fibers, high temperature, mechanical properties, cooling methods

## Abstract

The mechanical properties of cementitious composites before and after exposure to high temperature are affected by calcium–silicate–hydrate (C–S–H) gels. To evaluate the effects of high temperature, plyvinyl alcohol (PVA) fiber content, and the cooling method on properties of cementitious composites, physical, mechanical, and microscopic tests were performed in this study. The target temperatures were 25, 100, 200, 300, 400, 600, and 800 °C. The PVA fiber contents were 0.0, 0.3, 0.6, 0.9, 1.2, and 1.5 vol%. The high-temperature resistance of PVA fiber-reinforced cementitious composite (PVA-FRCC) specimens was investigated through changes in their appearance, mass loss, compressive strength, splitting tensile strength, flexural strength, and microstructure. The results showed that PVA fibers reduced the probability of explosion spalling in the PVA-FRCC specimens exposed to high temperatures. The mass loss rate of samples exposed to temperatures below 200 °C was small and lower than 5%, whereas a significant mass loss was observed at 200 °C to 800 °C. A small rise in the cubic compressive and splitting tensile strengths of samples was found at 400 °C and 300 °C, respectively. Below 400 °C, the fibers were beneficial to the mechanical strength of the PVA-FRCC specimens. Nevertheless, when the temperature was heated above 400 °C, melted fibers created many pores and channels, which caused a decrease in the strength of the specimens. The method of cooling with water could aggravate the damage to the cementitious composites exposed to temperatures above 200 °C. High temperature could lead to the decomposition of the C–S–H gels of the PVA-FRCC samples, which makes C–S–H gels lose their bonding ability. From the perspective of the microstructure, the structure of PVA-FRCC samples exposed to 600 °C and 800 °C became loose and the number of microcracks increased, which confirmed the reduction in macro-mechanical properties.

## 1. Introduction

Invented in the 1820s, cement has been studied for almost 200 years. Cementitious composites with cement as the primary material are extensively used in the construction of infrastructure, such as highways, bridges, dams, and houses [1]. They have become the most commonly used building material worldwide [2]. They possess the advantages of easy acquisition, high strength, convenient construction, and low cost, which make them popular in the engineering field. However, several hidden dangers are associated with the use of cementitious composites. While their compressive strength is excellent, their tensile performance is not satisfactory. The tensile strength of cementitious composite is only about 5–10% of its compressive strength. In addition, they possess low flexural strength, poor toughness, and weak deformation. Furthermore, they are heavy and prone to brittle failure without warning [3]. However, the actual failure of cementitious composite components and structures is not solely caused by external loads. Under the long-term action of natural environmental factors, the defects of cementitious composites are amplified, accelerating the aging, damage, and even failure of the components and structures, thus affecting their functionality and safety [4]. These issues considerably affect the maintenance cost and service life of infrastructure systems. Therefore, improving the properties of cementitious composites has become a focus in the engineering [5,6].

Fiber-reinforced cementitious composites (FRCCs) are composed of a hardened cement paste as the matrix mixed with non-continuous short fibers or continuous long fibers randomly distributed in the matrix as reinforcement materials. These composites overcome the deficiencies of traditional cementitious composites, namely, low tensile strength, poor toughness, high brittleness, and poor crack control. The properties of FRCCs were determined by numerous factors, such as strength of cement paste and type and content of fibers. Li [7] successfully proposed the design concept of engineered cementitious composites (ECCs) based on the principles of micromechanics in 1993. An ECC is a typical FRCC representative. Various fiber types (such as steel [8], carbon [9], polypropylene [10], glass [11], polyvinyl alcohol (PVA) [12], and basalt [9]) are used in the production of FRCCs. Compared to other fibers, PVA fibers have the advantages of good dispersibility, excellent hydrophilicity, high tensile strength, high elastic modulus, superior bonding properties with cementitious materials, and nontoxicity [13]. In addition, the good acid and alkali resistance of PVA fibers can ensure that the cement matrix is not easily eroded [14]. Therefore, the application of PVA fibers in FRCCs is promising.

Researchers from various countries have conducted numerous experiments to investigate the basic mechanical properties and durability of PVA-FRCCs [15,16]. Meng et al. [17] used PVA fibers to prepare a PVA fiber-reinforced ECC (PVA-ECC), which possessed good mechanical properties (For a typical PVA-ECC with fiber volume fraction of 2%, tensile strain capacity of 4% and ultimate strength of 4.5 MPa can be achieved) and met structural design requirements. Owing to its tight crack width and high tensile strength, the PVA-FRCC represents a new cementitious composite with great potential for effectively solving the durability problem of concrete structures [18]. In addition, the engineering performances of PVA-reinforced geopolymer composites [19] have been extensively studied. However, the applications of FRCCs are becoming more prevalent, owing to their excellent engineering properties, increasing their risk of exposure to high temperatures. For cementitious composites, high temperatures cause not only a change in appearance and mass, but also a reduction in mechanical properties [20]. The mechanical strength of cementitious composites is largely determined by the hydration products generated by the cementitious material and water. The free water in cementitious composites exposed to lower temperatures (100–200 °C) evaporates first. At 180–300 °C, a portion of the C–S–H gels is dehydrated. At 400–600 °C, calcium hydroxide undergoes dihydroxylation [21]. When the temperature is raised above 600 °C, calcium carbonate begins to decompose [22]. All of these changes decrease the mechanical strength of the cementitious composites. Moreover, melting of PVA fibers of FRCCs exposed to elevated temperatures results in the formation of pores and channels in the matrix, which further reduces the mechanical properties of FRCCs [12]. However, the presence of these pores and channels can release steam pressure and reduce the spalling of PVA-FRCCs [23].

Vejmelkova et al. [24] tested the physical performance and mechanical strength of cementitious composites containing hybrid PVA fibers at elevated temperatures. The results showed a significant increase in porosity and a reduction in tensile and flexural properties from room temperature to an elevated temperature of 600 °C. From the perspective of the microstructure, these changes were mainly caused by the decomposition of Ca(OH)_2_ and C–S–H gels at high temperatures. According to Tian et al. [25], the residual compressive and flexural strengths of cementitious composites containing 2% PVA fibers subjected to temperatures from 200 °C to 400, 600, and 800 °C decreased gradually. After exposure to 800 °C, the residual compressive strength and flexural strength of the specimens were only 37% and 17.3% of the original ones, respectively. The addition of PVA fibers accelerated the reduction rate of the flexural strength of cementitious composites at high temperatures [26,27]. However, high temperature had little effect on the mass loss rate of the specimens. When the temperature was increased from 0 °C to 800 °C, the mass loss rate of PVA-FRCC ranged from 13% to 15.8%. Furthermore, the thermal stability of mortar could be improved by the PVA fibers. The formation of a large number of pores, after the fibers were melted, reduced the probability of explosion spalling of the mortar at high temperatures [12,28]. Yu et al. [4] further investigated the impact of the cooling method on the performance of cementitious composites after exposure to elevated temperatures. They found that cooling with water was helpful for the recovery of the strength and stiffness of specimens.

Currently, most studies on PVA-FRCCs mainly focus on mechanical properties, durability, and microscopic mechanisms at room temperature [29]. However, fires have occurred frequently in recent years, and many researchers worldwide have conducted preliminary research on the high-temperature resistance of FRCCs [30,31]. There are only few relevant studies on the basic mechanical properties and damage mechanism of PVA-FRCCs exposed to high temperatures. Thus, it is necessary to further explore the high-temperature resistance of PVA-FRCCs.

This study analyzed the basic mechanical properties of PVA-FRCCs after high-temperature exposure and evaluates the influence of heating temperature, PVA fiber content, and cooling method on their mechanical properties.

## 2. Experimental Results and Discussions

### 2.1. Experimental Phenomena

**Figure 1:**
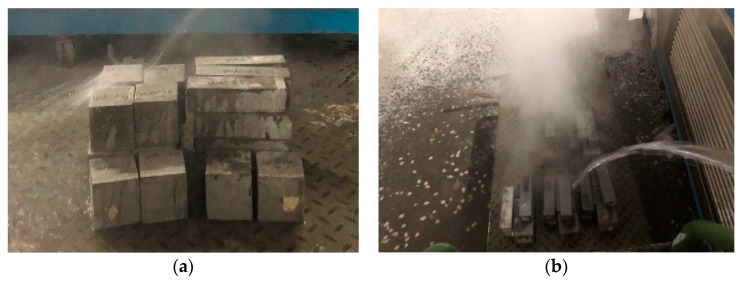
Cooling of specimens after high-temperature exposure. (**a**) 200 °C and (**b**) 600 °C.

Figure 1 shows the cooling of specimens after high-temperature exposure. A relatively small amount of steam, which disappeared after 3–4 min, was observed on the specimens subjected to temperatures of 200 °C and 400 °C during cooling with water. In contrast, a large amount of steam, which disappeared after 5–6 min, was produced on the specimens exposed to temperatures of 600 °C and 800 °C.

When the temperature reached approximately 200 °C, a small amount of visible steam began to emerge from the electric furnace door during the heating process, and an unpleasant odor, similar to that of burned plastic, was emitted. After the temperature was maintained at 200 °C, a small amount of water droplets seeped from the furnace mouth along the joint for some time. As the temperature reached 300–400 °C, the amount of steam increased significantly. Then, when the temperature was approximately 400 °C, a large amount of thick smoke, accompanied by a pungent smell, was emitted. This process lasted approximately 15 min and then the rate of thick smoke emission gradually decreased. However, a large number of water droplets accumulated on the upper edge of the furnace mouth. When the temperature was heated above 550 °C, the amount of steam gradually decreased; this lasted for a long time until the steam dissipated. In addition, when the temperature was increased to 400, 600, and 800 °C, a bursting sound from the electric furnace was heard. Some cementitious composite samples without PVA fibers experienced explosive spalling. In contrast, no explosive spalling occurred on the PVA-FRCC specimens during the entire duration of high-temperature exposure, indicating that PVA fibers help to resist explosive spalling.

The above high-temperature experimental phenomena can be explained by several aspects. When the temperature was 100–200 °C, the free water in the specimens began to evaporate because of the physical dehydration. When the temperature reached 300–500 °C, a large amount of free water and crystal water in the C–S–H gels broke away from the chemical bond and gradually escaped from the interior of the materials. When the temperature exceeded 550 °C, the free water and bonded water evaporated, and the hydration products began to decompose. The explosive spalling of the specimens without PVA fibers after exposure to high temperature is brittle failure, which can be explained by the vapor pressure theory. That is, due to the different speed of steam escape at different depths of specimens, a dry zone, a steam zone and a wet zone will be formed from shallow to deep inside the cementitious composites. When the steam pressure in the steam zone is greater than the tensile strength of the cementitious composites, the explosive spalling will occur. It can also be explained by the thermal dilation theory. That is, cementitious materials subjected to high temperatures will generate a temperature gradient from the outside to the inside. When the tensile stress caused by the uneven thermal expansion is greater than the tensile strength of cementitious material, the surface of material will burst and spall [32]. For cementitious composites with PVA fibers, the pores formed by the melted fibers after high-temperature exposure provide channels for the release of water vapor. At the same time, the pore network formed by the scattered fibers in the cementitious composites greatly increases the permeability of the material and slows down the rising rate of the vapor pressure of water, which effectively prevents explosive spalling [32,33]. Other researchers have obtained similar results [34,35].

### 2.2. Appearance Changes and Mass Loss after High-Temperature Exposure

**Figure 2:**
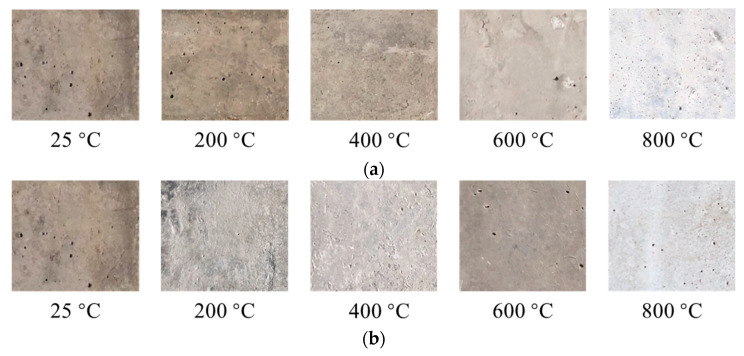
Color changes of specimens subjected to different cooling methods. (**a**) Cooling at room temperature. (**b**) Cooling with water.

It was found that the apparent changes in the samples with different proportions, after being subjected to elevated temperatures, were essentially the same. As the temperature increased, the surface color of the cementitious composite material gradually became shallow, and the number of cracks and spalling gradually increased. The samples began to crack, owing to dehydration and decomposition of the C–S–H gels and AFt when the temperature exceeded 400 °C. The detailed appearance changes of the specimens after elevated temperature exposure are presented in Table 1. Figure 2 shows the color changes of the specimens cooled at room temperature and those cooled with water after high-temperature exposure. Unusually, the color of samples cooled with water turned to dark gray after exposure to 600 °C, which may be attributed to the further hydration reaction of cementitious composites [4].

**Figure 3:**
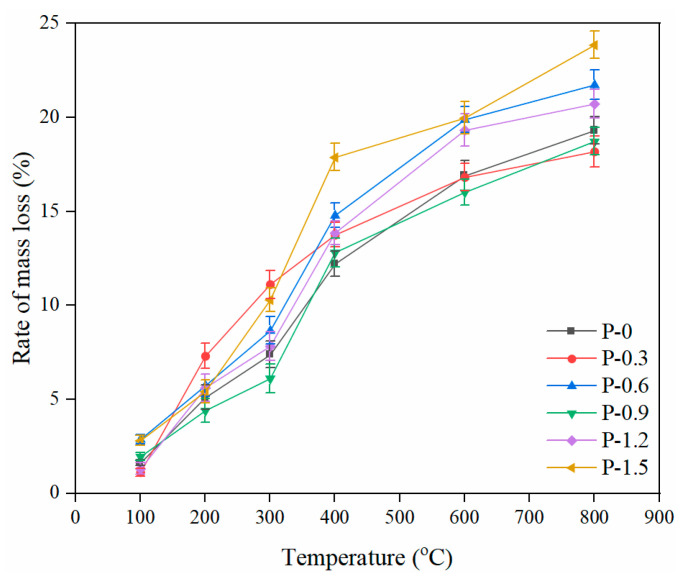
Mass loss rate of PVA-FRCC specimens after exposure to different temperatures.

The deterioration of the samples at high temperature was also assessed by the mass loss. Figure 3 depicts the relationship between the mass loss rate of PVA-FRCC samples and temperature. The mass loss rate of samples increases with the heating temperature. The mass loss is mainly related to the loss of various types of water in the matrix after high-temperature exposure. When the temperature is below 200 °C, the mass loss is mostly due to the loss of a small amount of free water. The mass loss rate is small and lower than 5%, and the growth rate is relatively gradual. When the temperature increases from 200 °C to 400 °C, large amounts of free water and chemical crystal water evaporate or decompose [36]. At this stage, the mass loss rate of specimens increases significantly, and the rate of change accelerates. The maximum mass loss of some of the specimens reaches 17.88% at 400 °C. When the temperature is between 400 °C and 600 °C, the mass loss is mostly caused by decomposition of Ca(OH)_2_ and bond loss of C–S–H gels [37]. In addition, the rate of increase in mass loss decreases significantly. The water inside the matrix evaporates, and carbonate begins to decompose when the heating temperature exceeds 600 °C.

The mass loss rate of the specimen with a PVA fiber content of 0.9% is slightly lower than that of reference specimen. When the PVA fiber content is 1.2% and 1.5%, the agglomeration of the fibers makes the mixing uneven, resulting in an increase in the internal porosity of the cementitious composite materials and rapid evaporation of water during the heating process. Consequently, their mass losses exceeded that of the control specimen. PVA fibers that were almost completely melted at 400 °C also had effect on mass loss [38]. In addition, the mass loss of the specimen containing 0.6% PVA fibers was higher than that of the specimen containing 0.9% PVA fibers, which may be due to the uneven dispersion of fibers caused by human error in the process of mixing cementitious composites.

### 2.3. Compressive Strength after High-Temperature Exposure

**Figure 4:**
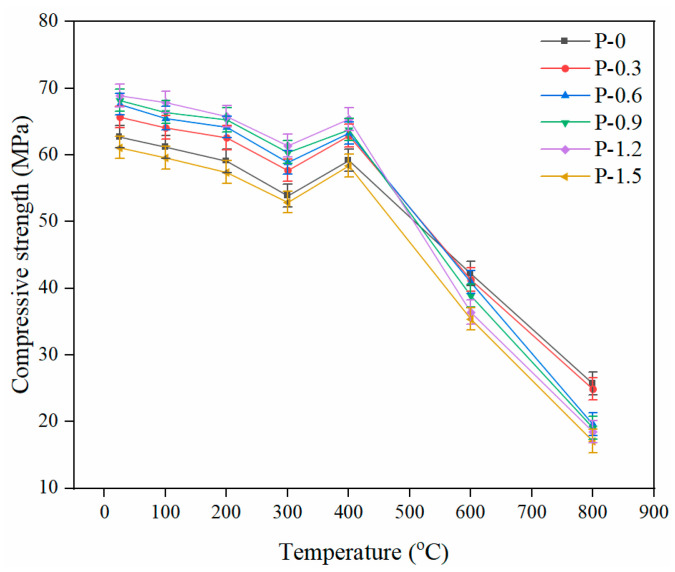
Compressive strength of PVA-FRCC specimens with different fiber contents.

Figure 4 shows the trend of cube compressive strength of PVA-FRCC samples containing different fiber contents as a function of temperature. The high temperature can cause a reduction in the cube compressive strength of specimens. When the exposure temperature of the cementitious composites was lower than 300 °C, the compressive strength gradually decreased with temperature. Additionally, when the exposure temperature of the cementitious composites exceeded 400 °C, the compressive strength decreased sharply with temperature. The minimum relative compressive strength of PVA-FRCC specimens is 85.9% at 300 °C, 52.8% at 600 °C and only 26.9% at 800 °C. The reason for the strength decline is that the free water evaporation or fiber melting produces pores and small microcracks when the temperature is below 300 °C [25,28]. Nevertheless, the compressive performance is not sensitive to microcracks; thus, the relative cubic compressive strength of each sample at 300 °C is still approximately 90%. As displayed in Figure 4, the compressive performance of PVA-FRCC specimens at 400 °C is better than that at 300 °C, and the increase rate is approximately 8%. In the process of free water evaporation, the cement gel layers are close to each other, resulting in greater van der Waals forces [39]. Further hydration of the cementitious materials leads to a denser structure of the PVA-FRCC samples. This is another significant reason for the strength increase. In particular, for cementitious materials containing FA, unhydrated FA particles promote the formation of C–S–H gels through a reaction with Ca(OH)_2_ [39]. While more pores and microcracks are induced by high temperature, this damage is completely overcome by the enhancement effect mentioned above. However, the compressive strength of specimens at 200 °C [4] or 250 °C [10] is higher than that at room temperature in other studies. Morsy et al. [40] concluded that the steam pressure inside the pores caused further hydration of unhydrated cement particles, which improved the compressive strength of the specimens. This phenomenon is known as the internal autoclaving influence.

**Figure 5:**
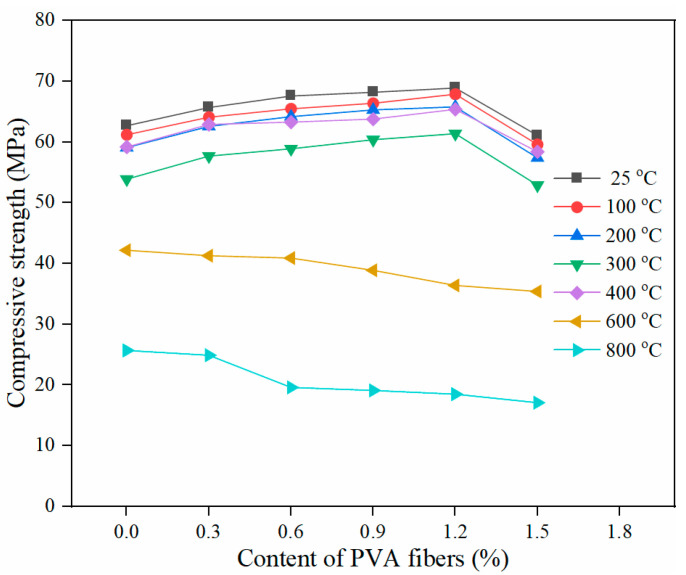
Compressive strength of PVA-FRCC samples after exposure to different temperatures.

Figure 5 presents the effect of PVA fiber content on the compressive strength of PVA-FRCC specimens after high-temperature exposure. The compressive performance of cementitious composites with fibers is better than that without fibers in the temperature range of 25–400 °C. With an increasing content of PVA fibers, the compressive strength at different temperatures increases first and then reduces. The compressive strength of the sample reaches the maximum when the PVA fiber content is 1.2%. At this condition, the compressive strengths are 68.9, 67.9, 65.8, and 61.4 MPa after exposure to 100, 200, 300, and 400 °C, respectively. These correspond to an increase of 9.9%, 10.9%, 11.3%, and 13.9%, respectively, as compared to the strength at room temperature. The compressive strength of PVA-FRCC samples after exposure to 600 °C and 800 °C reduces gradually with the PVA fiber content. In contrast to the reference group, the strength of the specimen with 1.5% fiber content decreases by 16.1% and 33.5%, respectively. An appropriate amount of PVA fibers can not only inhibit the generation and development of cracks, but also satisfactorily restrain the thermal deformation of the cement matrix before fiber melting, resulting in the reduction of damage in the matrix and improvement in strength [41]. However, when the content exceeds 1.5%, fiber agglomeration can generate more pores and defects, causing a decrease in strength. When the specimens are subjected to a high temperature of 400 °C, the fibers are completely melted. The higher the fiber content, the greater the number of pores and channels formed after fiber melting and the lower the strength.

**Figure 6:**
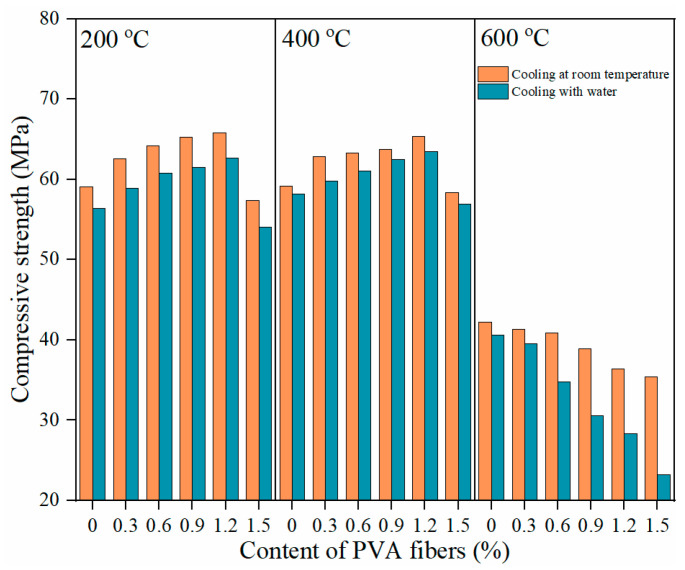
Compressive strength of PVA-FRCC specimens subjected to different cooling methods.

The above are the results of the compressive strength analysis of specimens cooled at room temperature after exposure to elevated temperatures. The compressive properties of PVA-FRCC samples cooled with water after exposure to 200, 400, and 600 °C were also investigated and compared to those cooled at room temperature. The compressive strengths of specimens subjected to different cooling methods are shown in Figure 6. After exposure to different high temperatures, the variation trend of compressive strength of the samples cooled with water is similar to that of the samples cooled in the natural state. However, the strength of the specimens cooled with water is less than that of the specimens cooled in the natural state. This indicates that cooling with water aggravates the high-temperature damage and reduces the strength [42]. Especially when the temperature is heated to 600 °C and the fiber content is 1.5%, the strength of sample cooled with water is 12.2 MPa lower than that of the sample cooled at room temperature. Moreover, the compressive strength of specimens cooled with water and sealed for seven days after exposure to 800 °C is much higher than that of specimens cooled at room temperature. Yu et al. [4] attributed this increase in strength to the generation of new crystals.

### 2.4. Splitting Tensile Strength after High-Temperature Exposure

**Figure 7:**
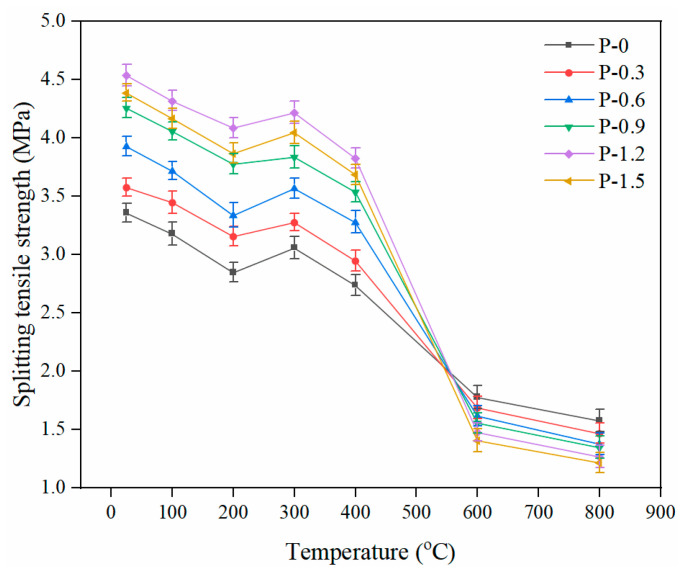
Splitting tensile strength of PVA-FRCC specimens with different fiber contents.

The tensile performance of FRCCs is extremely important for practical applications. Figure 7 depicts the trend of splitting the tensile strengths of the PVA-FRCC samples containing different fiber contents as a function of temperature. The tensile performance of specimens generally shows a decreasing trend with increasing temperature, which is similar to that of the compressive strength. The splitting tensile strength gradually decreases when the temperature is below 200 °C. As the temperature increases to 300 °C, the splitting tensile strength increases to a certain extent, but it is still lower than that of the sample at 25 °C. This trend differs from the improvement in compressive strength at 400 °C. In the range of 300–600 °C, the reduction rate of the splitting tensile strength increases significantly. Moreover, the strength continues to decrease from 600 °C to 800 °C, but the rate of decline decreases. Specifically, the average relative splitting tensile strength of the specimen at 200 °C is 87.5%, and the reduction is about 12.5%. The maximum increase of splitting tensile strength at 300 °C is 6.3%, as compared to that at 200 °C. With the increase in temperature, the splitting tensile strength of the specimen decreases significantly. The minimum relative strength of the specimen at 400 °C is 81.9% and the rate of strength is 18.1%. For the specimen exposed to 600 °C (the PVA fiber content is 1.5%), the rate of splitting strength loss reaches 67.9%, and the maximum loss rate of the specimen at 800 °C is 72.2%. A series of complex reactions occurred in the matrix after the specimens were subjected to high temperature [26]. After exposure to 200 °C, although some evaporated water caused microcracks and pores in the interior of the specimens, the unmelted fibers still had a bonding force and anti-cracking effect, leading to a gradual decline in tensile strength. A small increase in tensile strength at 300 °C is attributed to the secondary hydration reaction of water evaporation with unhydrated cement particles and the cracking resistance of the fibers. However, this strengthening effect is less than the damage effect caused by the high temperature on the splitting tensile strength. Therefore, its strength is still lower than that at 25 °C. At 300 °C, the evaporation of extensive water and melting of the PVA fibers increase the internal cracks in the matrix. When the temperature exceeds 400 °C, the decomposition of Ca(OH)_2_ and bonding loss of C–S–H gels intensify the internal deterioration, leading to a great decline in the splitting tensile strength of PVA-FRCC samples.

**Figure 8:**
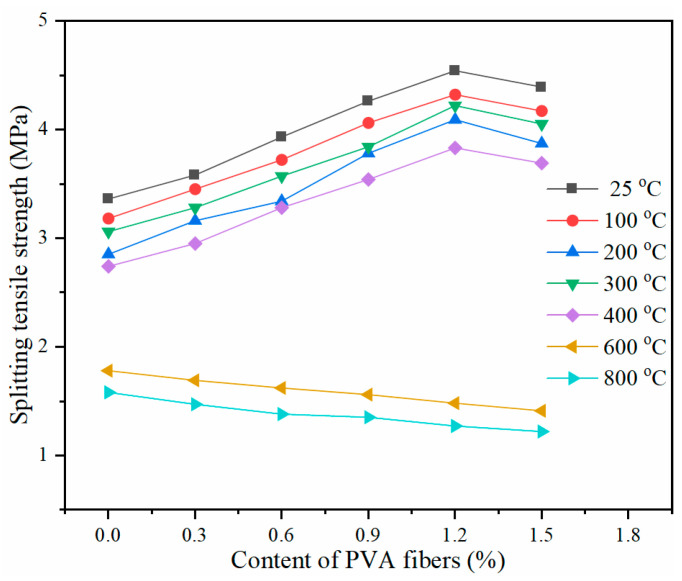
Splitting tensile strength of PVA-FRCC specimens after exposure to different temperatures.

Figure 8 illustrates the impact of the PVA fiber content on the splitting tensile strength of specimens subjected to high temperature. At temperatures below 400 °C, PVA fibers can enhance the splitting tensile property of the specimen. The variation of the splitting tensile strength with the PVA fiber content is similar to that of the compressive strength. At a 1.2% fiber content, the splitting tensile strength reaches the maximum. At 25, 200, 300, and 400 °C, the splitting tensile strengths of the specimens with 1.2% PVA fiber content are 35.1%, 43.5%, 37.9%, and 39.8% higher than that of the specimen without PVA fibers, respectively. When the temperature is elevated to 600 °C and 800 °C, the splitting tensile strength decreases with the increasing PVA fiber content because the fibers have completely melted and lost their bonds to the matrix.

### 2.5. Flexural Strength after High-Temperature Exposure

**Figure 9:**
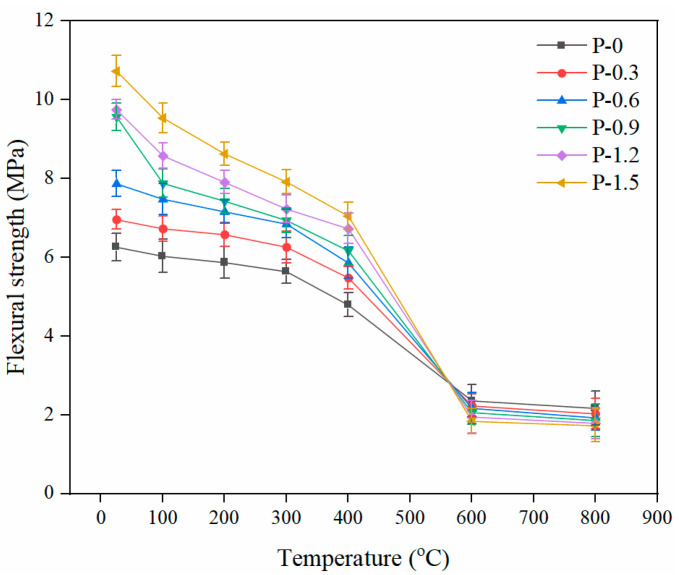
Flexural strength of PVA-FRCC specimens with different fiber contents.

The flexural strength is considered to be the ability of a concrete beam to withstand bending and destruction. Figure 9 shows the trend of the flexural strength of PVA-FRCC samples containing different fiber contents as a function of temperature. The flexural strength of PVA-FRCC specimens decreases monotonically with the heating temperature. The flexural strength declines gradually from 25 °C to 300 °C. The lowest relative flexural strength of 72.5% occurs at 300 °C. At 400 °C, the flexural strengths of the specimens are 4.79, 5.48, 5.86, 6.17, 6.73, and 7.05 MPa, respectively, and the maximum strength loss rate is 35.5% (at a PVA fiber content of 1.5%). The flexural strength decreases sharply at 400–600 °C, and the decrease is significantly higher than that at 300–400 °C. The average relative flexural strength at 600 °C is 25.9% and the maximum loss rate is 82.9%. The flexural strength decreases gradually after exposure to 600 °C. At 600–800 °C, the flexural strengths of the specimens are lower than that of the reference specimen, which indicates that the fibers do not have any beneficial influence on flexural strength in that temperature range [27].

In contrast to the compressive strength, the flexural strength did not increase slightly at 400 °C. This is because the flexural strength is more sensitive to crack damage than the compressive strength. While the secondary hydration of the cement made the matrix denser, it was not sufficient to overcome the damage caused by cracks and pores. In addition, the denser the matrix, the more brittle it becomes. As the temperature was elevated to 600 °C, the matrix became more sensitive to cracks. The PVA fibers then melted and lost the bridging effect, leading to a significant decrease in the flexural strength. When the temperature exceeded 600 °C, the internal structure of the PVA-FRCC specimens was severely damaged, making the flexural strength insensitive to cracks. Therefore, the flexural strength decreases at a slower rate.

**Figure 10:**
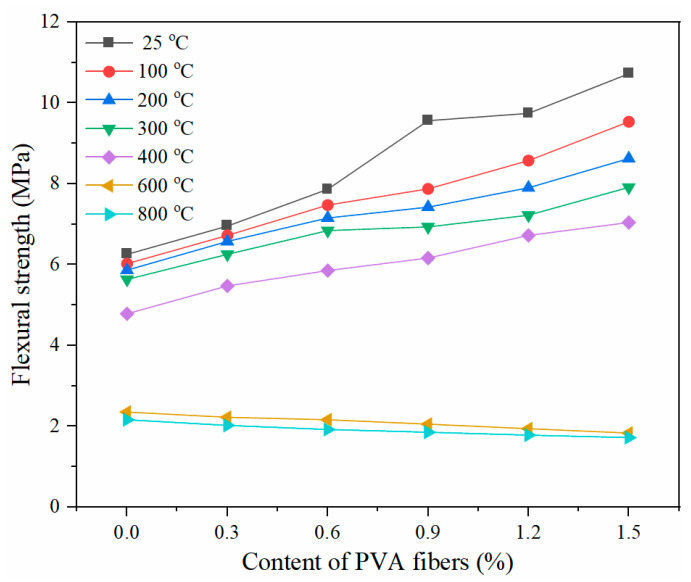
Flexural strength of PVA-FRCC after exposure to different temperatures.

Figure 10 illustrates the effect of PVA fiber contents on the flexural strength of the PVA-FRCC samples exposed to high temperature. When the temperature is below 400 °C, the flexural strength of the specimens increases with the increasing PVA fiber content. At a 1.5% PVA fiber content, the flexural strengths of the specimens at 25, 100, 200, 300, and 400 °C are 10.73, 9.54, 8.63, 7.92, and 7.05 MPa, respectively. These correspond to an increase of 71.4%, 58.2%, 47.0%, 40.4%, and 47.2%, respectively, compared to that of the specimen without fibers. The most significant increase in the flexural strength is at room temperature, and the higher the temperature, the lower the increase. This indicates that, when the PVA fibers are not completely melted, the influence of high temperature on them remains on the fiber surface. The fibers can provide a bridging effect at lower temperatures, thus improving the flexural strength. However, when the temperature reaches 600 and 800 °C, the completely melted fibers lose their bonds with the matrix, weakening the promotion impact of fibers on flexural strength. The larger fiber content corresponds to more defects and cracks that occur after high-temperature exposure, leading to a lower flexural strength [25].

**Figure 11:**
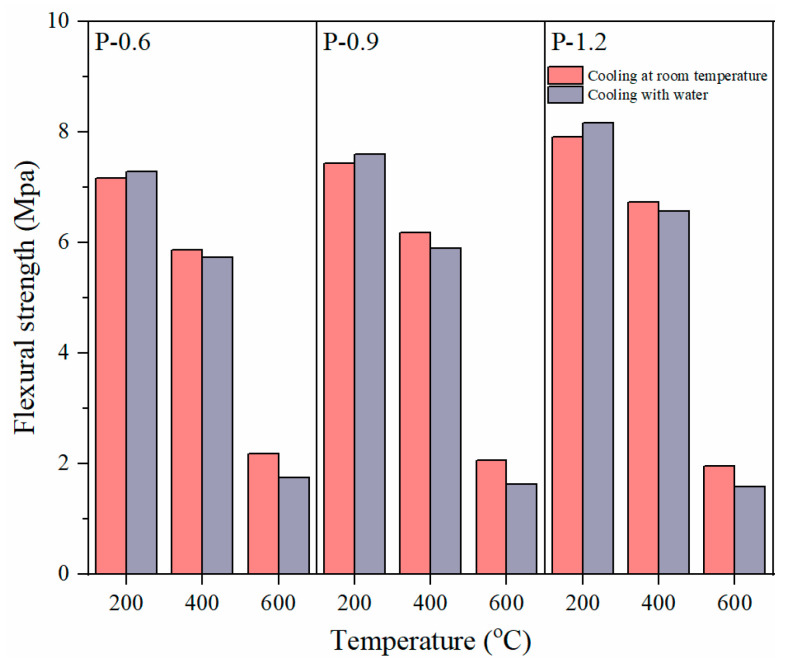
Flexural strength of PVA-FRCC specimens with different cooling methods.

The above results are from the flexural strength analysis of PVA-FRCC samples cooled under natural conditions after high-temperature exposure. Figure 11 depicts the variation trend of the flexural strength of the PVA-FRCC specimens with temperature under different cooling methods. The relationship between the flexural strength and temperature after cooling with water is the same as that after cooling at room temperature. At a 200 °C heating temperature, the flexural strengths of the PVA-FRCC samples cooled with water are 7.28, 7.59, and 8.16 MPa, which are 1.7%, 2.1%, and 3.2% higher than those of the specimens cooled at room temperature, respectively. However, as the temperature increases to 400 and 600 °C, the flexural strengths of the specimens cooled with water are lower than those of the specimens cooled at room temperature. Cooling with water causes more severe damage to the matrix exposed to 600 °C. A possible reason for this is that water can enter the specimen during water cooling for a period of time when the temperature is low, providing a humid environment for the secondary hydration reaction. This can compensate for the loss of internal structural damage caused by temperature stress and can improve the flexural strength. However, as the temperature exceeds 400 °C, the damage to the structure caused by the temperature is significant. The improvement caused by the secondary hydration reaction is considerably less than the damage caused by the high temperature. Therefore, the water-cooled specimens exhibited a lower flexural strength [43].

### 2.6. Microstructure Characterization

**Figure 12:**
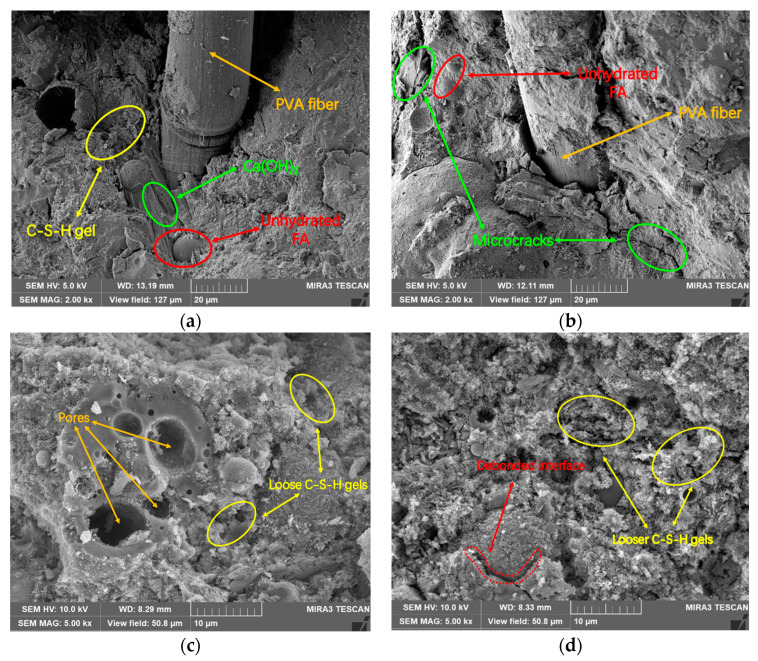
SEM micrographs of PVA-FRCC specimens subjected to different temperatures. (**a**) 25 °C, (**b**) 200 °C, (**c**) 400 °C, and (**d**) 600 °C.

To study the microstructure of the fibers and matrix at different temperatures, the specimens exposed to 200–800 °C were observed by the SEM. Figure 12 displays the micrographs of specimens with and without high-temperature exposure. The specimens at room temperature (Figure 12a) mainly consist of poorly crystallized and fibrous particles of C–S–H gels, amorphous and well-crystallized Ca(OH)_2_, and a large number of unhydrated FA particles. The dense structure in Figure 12a resulted in excellent mechanical properties of the specimens [2,44]. Compared to the microstructures of the unheated specimens, the specimens exposed to 200 °C did not change significantly. However, a few microcracks can be observed in Figure 12b, which mainly caused strength decline. Pores are clearly seen in Figure 12c. This is because, at 400 °C, the PVA fibers completely melted to form pores and channels. The existence of these pores and channels is conducive to the release of pore pressure, which significantly reduces the explosive spalling of the specimens. However, these pores and channels also significantly reduced the mechanical properties of the specimens. In addition, the loose microstructure can also be clearly observed. The microstructures of the specimens become looser after being exposed to 600 °C (Figure 12d) and 800 °C. All hydration products nearly lost their crystal structure characteristics. In general, C–S–H crystals can form disordered spaces in the matrix at high temperatures, leading to a reduction in microstructure densification [45]. Sahmaran [28] found that, when the temperature exceeds 800 °C, cracks around unhydrated FA particles increase, and quartz sands lose their characteristic structure.

## 3. Conclusions

The properties of PVA-FRCC specimens after exposure to high temperatures were investigated. The high-temperature resistance of the specimens was analyzed through changes in their appearance, mass loss, compressive strength, splitting tensile strength, flexural strength, and microstructure. By recording the experimental results, the following conclusions can be drawn:

(1) During the heating process, some samples of cementitious composites without PVA fibers burst. With the exposure temperature increasing, the appearance of color in the cementitious composite specimens gradually move from deep to shallow, and the number of cracks, spalling, and mass loss gradually increased. The final mass loss of the PVA-FRCC samples after exposure to 800 °C ranged from 18.19% to 23.86%.
(2) The high temperature reduced the compressive, splitting tensile strength and flexural strengths of the PVA-FRCC specimens. The mechanical properties of the PVA-FRCC samples decreased with increasing temperature. Before the PVA fibers were melted, the fibers were conducive to the tensile and flexural properties of the specimens. On the contrary, the pores and channels caused by melted fibers could significantly reduce the mechanical properties of specimens. In addition, the compressive strength of specimens cooled at room temperature exceeded that of the specimens cooled with water. The method of cooling with water could aggravate the damage caused by an elevated temperature.
(3) The dense microstructure of specimens resulted in excellent mechanical performance at room temperature. After exposure to 200 °C, the microstructure of the specimens did not change significantly. After exposure to 400 °C, the melted fibers caused the formation of many pores and channels, which primarily caused the strength decline. After being subjected to high temperatures of 600 °C and 800 °C, the microstructure became loose and the residual strength was reduced.

In this paper, the effect of heating temperature on the mechanical properties and microstructure of PVA-FRCC was investigated under the conditions of a fixed heating rate and constant temperature time. In the future, the influence of heating rate and constant temperature time on the specimens can be considered. In this paper, only SEM is used to observe the microstructure, and other characteristic methods of the microstructure are lacking. X-ray diffraction (XRD) should be further used to study the composition of hydration products and the atomic or molecular structure morphology of PVA-FRCC in future research. In addition, it is still necessary to pay attention to the research on the mechanical properties of PVA-FRCC exposed to open fire.

## 4. Experimental Program

### 4.1. Raw Materials

**Figure 13:**
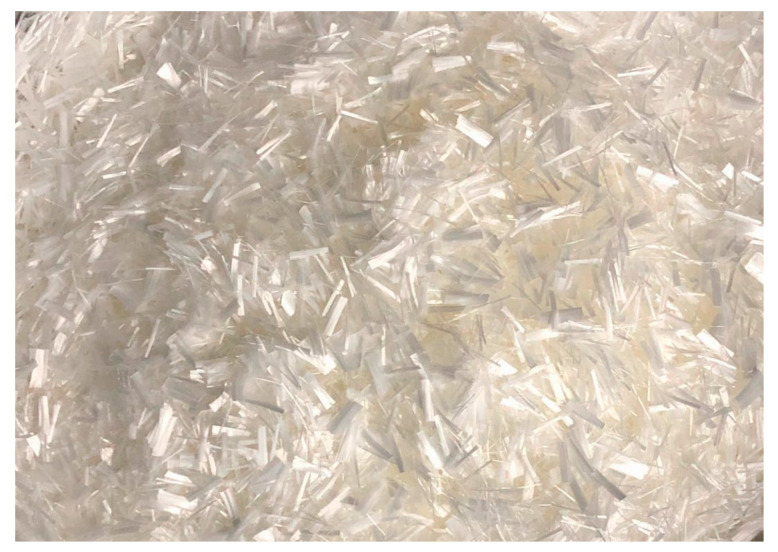
PVA fibers.

PVA-FRCC specimens were prepared from ordinary Portland cement (OPC) (in accordance with the common Portland cement standard (GB175-2007)) [46], Class I fly ash (FA), quartz sand, PVA fiber, water, and admixture. OPC was P.O 42.5. Table 2 presents the compositions of OPC and FA. The particle size of the quartz sand ranges between 75 μm and 120 μm. Table 3 provides the physical indices of the PVA fibers (Figure 13). A polycarboxylate acid superplasticizer (SP) was used as the admixture.

### 4.2. Mix Proportions and Preparation of PVA-FRCC Specimens

In the mix designs, the control variate method was adopted, and various PVA fiber contents were used. According to Zhang et al. [47], the water/cementitious material (W/B) ratio was 0.35, and the quartz sand/cementitious material (S/B) ratio was 0.5, after repeated tests and adjustments. When the W/B was unchanged, the volume contents of the PVA fibers were 0.3%, 0.6%, 0.9%, 1.2%, and 1.5%. The cementitious composites without PVA fibers are considered as reference groups. According to the principle of the workability consistency of each mixing proportion, the SP content, which was based on the cement content, was increased with increasing PVA fiber content. Table 4 shows the detailed mix proportions of PVA-FRCC specimens. There are six series in total, and 81 specimens are casted for each series.

The mixtures were stirred in a mechanical mixer with a capacity of 50 L. First, OPC, FA, and quartz sand were blended for approximately 1 min under dry conditions. Then, a premix of pure water and SP was poured and blended for 2 min. The PVA fibers were gradually added during the mixing process, and the mixture was blended for 2 min. Finally, the freshly blended mixtures were placed into molds.

### 4.3. Curing and Heating Regimens

**Figure 14:**
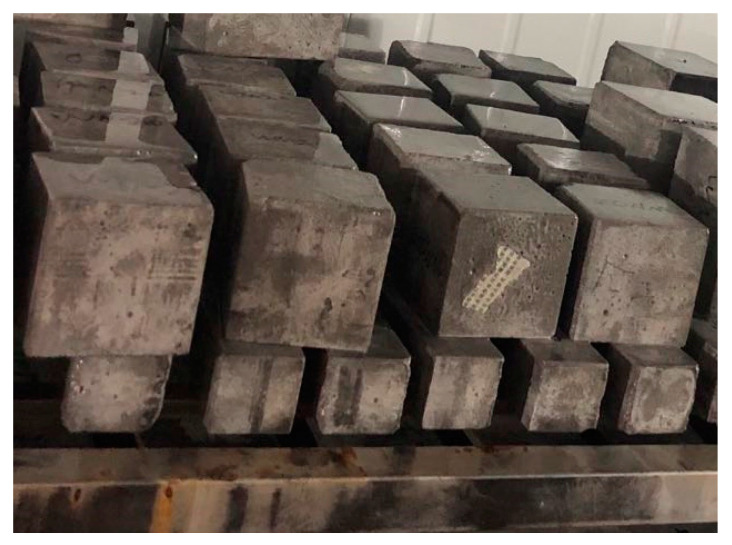
Specimens in the curing room.

After casting, the surfaces of the specimens were covered with a layer of plastic film. After 24 h, the specimens were removed from the molds [48]. The specimens were placed in a curing room (temperature 20 ± 2 °C, relative humidity ≥ 95%) for 28 days (Figure 14) and then taken out. To reduce the moisture content of the specimens to that in a normal state, they were placed in a dry and ventilated environment for 30 d. The specimens were then heated to the target temperature in a high-temperature electric furnace. The target temperature was then maintained for 120 min. Subsequently, the test specimens were removed and cooled until a constant temperature was reached. Two cooling regimens were adopted, namely, cooling at room temperature and water cooling. Relevant mechanical property tests were carried out after storing the specimens indoors for 3 d. The target temperatures were set as 25, 100, 200, 300, 400, 600, and 800 °C. The average heating rate was 5 °C/min. In addition, during the heating process, a certain gap between the specimens was set to prevent mutual influence [49].

### 4.4. Physical and Mechanical Property Tests

The appearance and mass of the specimens were recorded before and after exposure to elevated temperatures. Subsequently, their mass losses were calculated. The test specimens were cubes with dimensions of 100 × 100 × 100 mm. Three specimens were tested under each working condition and the average value was used as the test result.

The cube compressive and splitting tensile strengths were tested according to the test method of performance on building mortar (JGJ/T70-2009) [50]. The test specimens were cubes of 70.7 × 70.7 × 70.7 mm. The flexural strength was tested in accordance with the test method for the mechanical properties of mortar for ferrocement (GB/T 7897-2008) [51]. The test specimens were prisms of 40 × 40 × 160 mm. Three specimens were tested under each working condition, and the average value was used as the test result.

### 4.5. Microscopic Test

A scanning electron microscope (SEM, KYKY-EM6200) was utilized to examine the microstructure of PVA-FRCC samples. Pieces of less than 1 cm^3^ were dried in a furnace at 60 °C and sprayed with gold on the surface before the test.

## Figures and Tables

**Table 1:** Appearance changes of specimens after high-temperature exposure.

Temperature (°C)	Appearance Color		Crack State		Spalling
	Cooling at Room Temperature	Cooling with Water	Cooling at Room Temperature	Cooling with Water	
25	Dark gray	Dark gray	None	None	None
200	Dark gray	Gray	None	Few	None
400	Gray	Gray	Very few and thin	Many	None
600	Gray	Dark gray	Few and thin	All over	Few
800	Light gray	Light gray	Many	All over	Few

**Table 2:** Composition of OPC and FA.

Composition (%)	SiO_2_	Al_2_O_3_	Fe_2_O_3_	CaO	MgO	Na_2_O	K_2_O	SO_3_
OPC	21.05	5.28	2.57	63.14	3.58	0.17	0.58	2.39
FA	52.12	17.86	6.57	9.12	3.26	2.38	2.05	0.23

**Table 3:** Physical indices of the PVA fibers.

Length (mm)	Elongation (%)	Section Expansion Ratio (%)	Dry Elongation at Break (%)	Tensile Strength (MPa)	Alkali Resistance (%)	Melting Point (°C)
12	6.5	320	17	1540	99	230

**Table 4:** Mix proportions of PVA-FRCC specimens.

Mixture ID	OPC	FA	Quartz Sand	PVA Fiber	Water	SP
	kg/m^3^	kg/m^3^	kg/m^3^	%	kg/m^3^	kg/m^3^
P-0	650	350	500	0	350	1.5
P-0.3	650	350	500	0.3	350	2.0
P-0.6	650	350	500	0.6	350	2.5
P-0.9	650	350	500	0.9	350	3.0
P-1.2	650	350	500	1.2	350	3.5
P-1.5	650	350	500	1.5	350	4.0

## Data Availability

Not applicable.

## References

[1] Wang L., Li G., Li X., Guo F., Tang S., Lu X., Hanif A. (2022). Influence of reactivity and dosage of MgO expansive agent on shrinkage and crack resistance of face slab concrete. Cem. Concr. Compos..

[2] Golewski G.L., Szostak B. (2021). Strengthening the very early-age structure of cementitious composites with coal fly ash via incorporating a novel nanoadmixture based on C-S-H phase activators. Constr. Build. Mater..

[3] Ramachandra Murthy A., Karihaloo B.L., Vindhya Rani P., Shanmuga Priya D. (2018). Fatigue behaviour of damaged RC beams strengthened with ultra high performance fibre reinforced concrete. Int. J. Fatigue.

[4] Yu J., Weng W., Yu K. (2014). Effect of different cooling regimes on the mechanical properties of cementitious composites subjected to high temperatures. Sci. World J..

[5] Peng Y.X., Tang S.W., Huang J.S., Tang C., Wang L., Liu Y.F. (2022). Fractal analysis on pore structure and modeling of hydration of magnesium phosphate cement paste. Fractal Fract..

[6] Wang L., Yu Z.Q., Liu B., Zhao F., Tang S.W., Jin M.M. (2022). Effects of fly ash dosage on shrinkage, crack resistance and fractal characteristics of face slab concrete. Fractal Fract..

[7] Li V.C. (1993). From micromechanics to structure engineering-the design of cementitious composites for civil engineering application. JSCE.

[8] Caverzan A., Cadoni E., di Prisco M. (2013). Dynamic tensile behaviour of high performance fibre reinforced cementitious composites after high temperature exposure. Mech. Mater..

[9] Jogl M., Kotatkova J., Reiterman P. (2016). Differences in the mechanical properties of lightweight refractory cementitious composites reinforced by various types of fibers. Key. Eng. Mater..

[10] Alani S.S., Hassan M.S., Jaber A.A. (2019). Iop, Residual Strength and Degradation of Cement Mortar Containing Polypropylene Fibers at Elevated Temperature.

[11] Zhang W., Zhang Y., Wu Z., Liu N., Yuan D. (2019). Optimization design and properties of glass fiber reinforced cementitious composites. Mater. Rev..

[12] Çavdar A. (2012). A study on the effects of high temperature on mechanical properties of fiber reinforced cementitious composites. Compos. Part B Eng..

[13] Qian G., Dao X., Qian C. (2010). Effects of PVA fiber on mechanical property of concrete. China Concr. Cem. Prod..

[14] Zhang P., Gao Z., Wang J., Guo J.J., Wang T.Y. (2022). Influencing factors analysis and optimized prediction model for rheology and flowability of nano-SiO_2_ and PVA fiber reinforced alkali-activated composites. J. Clean Prod..

[15] Li V.C., Wang S.X., Wu C. (2001). Tensile strain-hardening behavior of polyvinyl alcohol engineered cementitious composite (PVA-ECC). ACI Mater. J..

[16] Tosun-Felekoglu K., Felekoglu B. (2013). Effects of fiber-matrix interaction on multiple cracking performance of polymeric fiber reinforced cementitious composites. Compos. Part B Eng..

[17] Meng D., Huang T., Zhang Y.X., Lee C.K. (2017). Mechanical behaviour of a polyvinyl alcohol fibre reinforced engineered cementitious composite (PVA-ECC) using local ingredients. Constr. Build. Mater..

[18] Gao S.L., Qi L., Wang W.C., Hu G.H., Shi H.F., Alhaj A. (2021). Fatigue fracture investigation of engineered cementitious composites. J. Test. Eval..

[19] Al-Majidi M.H., Lampropoulos A.P., Cundy A.B., Tsioulou O.T., Al-Rekabi S. (2018). A novel corrosion resistant repair technique for existing reinforced concrete (RC) elements using polyvinyl alcohol fibre reinforced geopolymer concrete (PVAFRGC). Constr. Build. Mater..

[20] Georgali B., Tsakiridis P.E. (2005). Microstructure of fire-damaged concrete. A case study. Cem. Concr. Compos..

[21] Durgun M.Y., Özen S., Karakuzu K., Kobya V., Bayqra S.H., Mardani-Aghabaglou A. (2022). Effect of high temperature on polypropylene fiber-reinforced mortars containing colemanite wastes. Constr. Build. Mater..

[22] Varona F.B., Baeza F.J., Bru D., Ivorra S. (2018). Evolution of the bond strength between reinforcing steel and fibre reinforced concrete after high temperature exposure. Constr. Build. Mater..

[23] Erdem T.K. (2014). Specimen size effect on the residual properties of engineered cementitious composites subjected to high temperatures. Cem. Concr. Compos..

[24] Vejmelkova E., Konvalinka P., Padevet P., Kopecky L., Keppert M., Cerny R. (2009). Mechanical, hygric, andthermal properties of cement-based composite with hybrid fiber reinforcement subjected to high temperatures. Int. J. Thermophys..

[25] Tian L., Zhang J., Dong S., Yuan G., Zahng Q. (2011). Study on mechanical properties of cementitious composites reinforced with PVA fibers after exposure to high temperatures. Concrete.

[26] Du Q., Wei J., Lv J. (2018). Effects of high temperature on mechanical properties of polyvinyl alcohol engineered cementitious composites (PVA-ECC). Int. J. Civ. Eng..

[27] Pourfalah S. (2018). Behaviour of engineered cementitious composites and hybrid engineered cementitious composites at high temperatures. Constr. Build. Mater..

[28] Şahmaran M., Özbay E., Yücel H.E., Lachemi M., Li V.C. (2011). Effect of fly ash and PVA fiber on microstructural damage and residual properties of engineered cementitious composites exposed to high temperatures. J. Mater. Civ. Eng..

[29] Zhang P., Wang W., Lv Y., Gao Z., Dai S. (2022). Effect of polymer coatings on the permeability and chloride ion penetration pesistances of nano-particles and fibers-modified cementitious composites. Polymers.

[30] Alakara E.H., Sevim O., Demir I., Simsek O. (2022). Experimental study on firebrick powder-based cementitious composites under the effect of elevated temperature. J. Build. Eng..

[31] Slusarek J., Nowoswiat A., Olechowska M. (2022). Logistic model of phase transformation of hardening concrete. Materials.

[32] Ozawa M., Morimoto H. (2014). Effects of various fibres on high-temperature spalling in high-performance concrete. Constr. Build. Mater..

[33] Ezziane M., Molez L., Messaoudene I. (2018). Non-destructive characteristion of mortars reinfored with various fibres exposed to high temperature. Min. Sci..

[34] Mueller P., Novak J., Holan J. (2019). Destructive and non-destructive experimental investigation of polypropylene fibre reinforced concrete subjected to high temperature. J. Build. Eng..

[35] Ezziane M., Kadri T., Molez L., Jauberthie R., Belhacen A. (2015). High temperature behaviour of polypropylene fibres reinforced mortars. Fire Saf. J..

[36] Zhang P., Han Q., Wu J., Zhang Y. (2022). Mechanical properties of nano-SiO_2_ reinforced engineered cementitious composites after exposure to high temperatures. Constr. Build. Mater..

[37] Ma Q., Guo R., Zhao Z., Lin Z., He K. (2015). Mechanical properties of concrete at high temperature—A review. Constr. Build. Mater..

[38] Shie J.L., Chen Y.H., Chang C.Y., Lin J.P., Lee D.J., Wu C.H. (2002). Thermal pyrolysis of poly(vinyl alcohol) and its major products. Energy Fuels.

[39] Khoury G.A. (1992). Compressive strength of concrete at high-tempertures-a reassessment. Mag. Concr. Res..

[40] Morsy M.S., Al-Salloum Y.A., Abbas H., Alsayed S.H. (2012). Behavior of blended cement mortars containing nano-metakaolin at elevated temperatures. Constr. Build. Mater..

[41] Yu K.Q., Dai J.G., Lu Z.D., Leung C.K.Y. (2015). Mechanical properties of engineered cementitious composites subjected to elevated temperatures. J. Mater. Civ. Eng..

[42] Chen Z., Zhou J., Wang C., Liu J. (2020). Experimental study on axial compression performance of recycled concrete filled square steel tube short column after exposure to high temperature and water cooling. J. Nat. Disasters..

[43] Han Q.Y., Zhang P., Wu J.J., Jing Y.T., Zhang D., Zhang T.H. (2022). Comprehensive review of the properties of fly ash-based geopolymer with additive of nano-SiO_2_. Nanotechnol. Rev..

[44] Yang H.M., Zhang S.M., Wang L., Chen P., Shao D.K., Tang S.W., Li J.Z. (2022). High-ferrite Portland cement with slag: Hydration, microstructure, and resistance to sulfate attack at elevated temperature. Cem. Concr. Compos..

[45] Kalifa P., Chene G., Galle C. (2001). High-temperature behaviour of HPC with polypropylene fibres—From spalling to microstructure. Cem. Concr. Res..

[46] (2007). Common Portland Cement.

[47] Zhang P., Wei S., Wu J., Zhang Y., Zheng Y. (2022). Investigation of mechanical properties of PVA fiber-reinforced cementitious composites under the coupling effect of wet-thermal and chloride salt environment. Case Stud. Constr. Mater..

[48] Danish A., Ozbakkaloglu T. (2022). Greener cementitious composites incorporating sewage sludge ash as cement replacement: A review of progress, potentials, and future prospects. J. Clean Prod..

[49] Zhang P., Han X., Hu S., Wang J., Wang T. (2022). High-temperature behavior of polyvinyl alcohol fiber-reinforced metakaolin/fly ash-based geopolymer mortar. Compos. Part B Eng..

[50] (2009). Standard for Test Method of Performance on Building Mortar.

[51] (2008). Test Methods of Mechanical Properties of Mortar for Ferrocement.

